# On periodic solutions of a discrete Nicholson’s dual system with density-dependent mortality and harvesting terms

**DOI:** 10.1186/s13662-021-03521-7

**Published:** 2021-07-31

**Authors:** Rajendiran Eswari, Jehad Alzabut, Mohammad Esmael Samei, Hui Zhou

**Affiliations:** 1 Department of Mathematics, Bharathidasan University, Tiruchirappalli, 620 024 Tamil Nadu, India; 2 Department of Mathematics and General Sciences, Prince Sultan University, Riyadh, Saudi Arabia; 3 Department of Industrial Engineering, OSTIM Technical University, 06374 Ankara, Turkey; 4 Department of Mathematics, Bu-Ali Sina University, Hamedan, Iran; 5 Department of Medical Research, China Medical University Hospital, China Medical University, Taichung, Taiwan; 6 School of Mathematics, Hefei Normal University, Hefei, 230039 P.R. China

**Keywords:** 34L30, 47H11, 54H25, Discrete Nicholson’s system, Variable delays, Continuation theorem, Periodic solution

## Abstract

In this study, we discuss the existence of positive periodic solutions of a class of discrete density-dependent mortal Nicholson’s dual system with harvesting terms. By means of the continuation coincidence degree theorem, a set of sufficient conditions, which ensure that there exists at least one positive periodic solution, are established. A numerical example with graphical simulation of the model is provided to examine the validity of the main results.

## Introduction

Global stability means that the attracting basin of trajectories of a dynamical system is either the state space or a certain region in the state space, which is the defining region of the state variables of the system. In other words, global stability means that any trajectories finally tend to the attractor of the system, regardless of initial conditions. For most of biological systems, population dynamics, e.g., gene regulatory systems, are needed to be globally stable [1–4]. In addition, the global asymptotical stability of the positive equilibrium of a dynamical system is one of the research foci in theoretical studies of both continuous and discrete bio-mathematical models [5–11].

Long investigated a patch structure Nicholson’s blowflies model involving multiple pairs of different time-varying delays

$$\begin{aligned} k'_{i} (t) = -\alpha _{ii} k_{i}(t) + \sum_{j=1,j\neq i}^{n} \alpha _{ij} k_{j}(t) + \sum_{j=1}^{n} \beta _{ij} (t) k_{i}\bigl(t-f_{ij}(t)\bigr) e^{- \gamma _{ij} (t) k_{i}(t- g_{ij}(t))} \end{aligned}$$

for $t \geq t_{0}$, $i=1,2,\dots,n$ [11]. The author established three novel criteria to check the global convergence, generalized exponential convergence, and asymptotical stability on the zero equilibrium point of the addressed model, without assuming the uniform positiveness of the death rate and the boundedness of coefficients, respectively (see [11]). Zhang *et al.* established the existence and global exponential stability of positive almost periodic solutions for Nicholson’s blowflies systems involving patch structure and nonlinear density-dependent mortality terms by applying differential inequality techniques and the fluctuation lemma which guarantees the existence of positive asymptotically almost periodic solutions for the addressed system [12]. Qian *et al.*, under some assumptions, developed a novel approach to demonstrate the global stability of positive asymptotically almost periodic solutions for the nonlinear density-dependent mortality Nicholson’s blowflies system

$$\begin{aligned} k'_{i} (t) ={}& {-}a_{ii} (t) + b_{ii} (t) e^{-k_{i}(t)} + \sum_{j=1,j \neq i}^{n} \bigl( a_{ij} (t)- b_{ij}(t) e^{ - k_{j}(t)} \bigr) \\ &{} + \sum_{j=1}^{n} \beta _{ij} (t) k_{i}\bigl(t-f_{ij}(t)\bigr) e^{- \gamma _{ij} (t) k_{i}(t- f_{ij}(t))} \end{aligned}$$

for $i=1,2,\dots, n$ [13]. Sweilam *et al.* considered variable-order fractional coupled nonlinear Burger’s equations under proportional delay *a*, *b*, and *c* in two dimensions (2-D) with the Atangana–Baleanu–Caputo (ABC) derivatives as follows:

$$\begin{aligned} &{}^{ABC}\mathcal{D}_{t}^{\alpha (x, y, t)} u_{t}(x, y, t) + \lambda _{1} u(ax, by, ct) u_{x}(x, y, ct) + \beta _{1} v(x, y, t) u_{y} (x, y, t) \\ &\quad = \rho \bigl(u_{xx} (x, y, t) + u_{yy} (x, y, t)\bigr), \\ &{}^{ABC}\mathcal{D}_{t}^{\alpha (x, y, t)} v_{t}(x, y, t) + \lambda _{2} u(ax, by, ct) v_{x}(x, y, ct) + \beta _{2} v(x, y, t) v_{y} (x, y, t) \\ &\quad = \rho \bigl(v_{xx} (x, y, t) + v_{yy} (x, y, t)\bigr), \end{aligned}$$

with the initial conditions $u(x, y,t_{0} ) = g_{1}(x,y), v(x, y, t_{0})= g_{2}(x, y)$, $x, y \in [L_{0}, L]$, and some boundary conditions where $0 < \alpha x, y, t)\leq 1$ and $u(x, y, t)$ and $v(x, y, t) $ are velocity components, *ρ* is a diffusion coefficient, $\lambda _{1}$, $\lambda _{2}$, $\beta _{1}$, and $\beta _{2}$ are constants, $g_{1}(x, y)$, $g_{2}(x, y)$, $f_{1}(x, y, t)$, and $f_{2}(x, y, t)$ are all known functions, $t_{0} $ is the initial time, *a*, *b*, $c \in (0,1)$ [3]. Also, Sweilam *et al.* in [7] investigated the effect of the optimal control of the variable order for HIV/AIDS and malaria mathematical models with multi-time delay and developed an efficient numerical algorithm to approximate the solutions of the proposed model with three control variables to reduce the number of the infected individuals of malaria and HIV/AIDS, and presented the numerical simulations for the obtained variable-order fractional system. Jaradat *et al.* considered the effect of inherited memory time and delay time in the formulation of a mathematical population growth model

$$\begin{aligned} \mathcal{D}_{t}^{\alpha }P(x, t) - \mathcal{D}_{xx} \bigl(P^{2}(x, t) \bigr) - aP(x, \tau t) + b P^{2}(x, \tau t)=0 \end{aligned}$$

for $t >0$, where $\alpha \in (0,1]$ is the Caputo derivative, and introduced two different numerical schemes to study analytically the propagation of population growth [6].

In 1986, Freedman *et al.* introduced criteria, such as population dynamics that has always been a core topic in theoretical ecology, which are established for three classes of models of single-species dynamics with a single discrete delay to have a globally asymptotically stable positive equilibrium independent of the length of delay [5]. In fact, asymptotic mean square stability of the linear part of the considered equation is used to verify stability in probability of nonlinear origin equation. In biological applications, a recruitment-delayed model

$$\begin{aligned} \frac{{\mathrm{d}} k}{{\mathrm{d}} t} = \mathcal{B}\bigl(k(t - t_{1})\bigr) - \mathcal{D}\bigl(k(t)\bigr) \end{aligned}$$

is frequently used, where $k(t)$ is a population size, the birth function $\mathcal{B}$ involves maturation delay $t_{1}$, and the death rate $\mathcal{D}$ depends on the current population level only. In order to characterize the population of the Australian sheep blowflies and to coincide well with the experimental result, Nicholson [14] and Gurney [15] introduced Nicholson’s blowflies model

$$\begin{aligned} k'(t)= - a k(t) + b k(t-t_{1}) \exp \bigl( - c k( t - t_{1}) \bigr), \end{aligned}$$

where $k(t)$ denotes the population size at time *t*, *a* is the per capita daily adult death rate, *b* denotes the maximum per capita daily egg production, $\frac{1}{c}$ is the size at which the blowfly population reproduces at its maximum rate, and $t_{1}$ is delay of the generation time. In 2015, Deng *et al.* solved an open problem on the global attractivity of the following diffusive Nicholson’s blowflies equation with distributed delay:

$$\begin{aligned} y_{t} - d \Delta y ={}& {-} \lambda y(x, t) + \alpha \tau \biggl( \int _{- \infty }^{t} w(t- \xi ) y(x, \xi ) {\mathrm{d}}\xi \biggr) \\ &{} \times \exp \biggl(- \int _{-\infty }^{t} w(t- \xi ) y(x, \xi ) {\mathrm{d}}\xi \biggr) \end{aligned}$$

for $(x, t) \in \Upsilon \times (0, \infty )$, where ϒ is a bounded domain in $\mathbb{R}^{N}$ with smooth boundary *∂*ϒ, the parameters *α*, *λ* are positive constants, and nonnegative kernel function *w* satisfies $\int _{0}^{\infty }w(\xi ) {\mathrm{d}}\xi =1$ [16]. In 2019, Gao *et al.* studied the conformable $(2+1)$-dimensional Ablowitz–KaupNewell–Segur equation in order to show the existence of complex combined dark-bright soliton solutions [12]. To this purpose, an effective method, i.e., the sine-Gordon expansion method, was used [12]. The 2D and 3D surfaces under some suitable values of parameters were also plotted [12].

Recently, many researchers have worked on a numerical technique to solve some types of equations such as Burgers’ equations with proportional delay, mathematical models with multi-time delay, an SEIR epidemic model for COVID-19 transmission, and biomathematics model inherited with memory time and delay time [3, 6, 7, 17]. In 2020, Yel *al.* employed the sine-Gordon expansion method to shallow water wave models which are Kadomtsev–Petviashvili–Benjamin–Bona–Mahony and the Benney–Luke equations [18]. They constructed many new complex combined dark-bright soliton, anti-kink soliton solutions for the governing models, and the 2D, 3D, and contour plots were given under the suitable coefficients [18]. Also, García Guirao *et al.* applied the sine-Gordon expansion method to the extended nonlinear $(2+1)$-dimensional Boussinesq equation [10]. Many new dark, complex, and mixed dark-bright soliton solutions of the governing model have been derived [10]. Moreover, for better understanding of the results, 2D, 3D, and contour graphs under the strain conditions and the suitable values of parameters were also plotted [10]. In 2018, the authors considered a discrete Nicholson’s blowflies model

$$\begin{aligned} \Delta k(n) = - \alpha (n) k(n) + b(n) + \beta (n) \ln k\bigl(n-\tau (n)\bigr). \frac{k(n)}{k^{\gamma (n)} (n - \tau (n))}, \end{aligned}$$

where $n \in \mathbb{Z}$ and $\alpha, b, \beta, \gamma, \tau: \mathbb{Z} \to [0, \infty )$ are almost periodic sequences, which involve a nonlinear density-dependent mortality term, and by using a fixed point theorem and Lyapunov functional method, obtained the existence and locally exponential stability of pseudo almost periodic solutions for the addressed Nicholson’s blowflies model [19].

In [20], Berezansky *et al.* focused on a linear model of density-dependent mortality terms

$$\begin{aligned} P'(t) = a P(t-t_{1})\exp \bigl( -c P(t-t_{1}) \bigr) - \mathcal{D}[P](t), \end{aligned}$$

where $\alpha >0$ and the form of function $\mathcal{D}$ may be $\mathcal{D}[P] = \frac{ mP}{ P + n}$ or $\mathcal{D}[P]= m - n\exp (-P)$, with positive constants $m>0$ and $n>0$. In 2017, the authors considered the non-autonomous almost periodic Nicholson’s blowflies model with density-dependent mortality term of the form

$$\begin{aligned} k'(t) = -\frac{a(t)k(t)}{b(t)+ k(t) } + p(t) k\bigl(t-\tau (t)\bigr) e^{-\beta (t) k(t - \tau (t))}, \end{aligned}$$

where $a(t)$, $b(t)$, $\beta (t)$, $p(t)$, and $\tau (t) \in C(\mathbb{R}, \mathbb{R}^{+})$ and $a(t)$, $b(t)$, $\beta (t)$, $p(t)$, $\tau (t)$ are bounded almost periodic functions [21]. Also many authors extensively studied Nicholson’s blowflies model with density-dependent mortality term (see for example [22–24]). Incorporating the phenomena gives us impulsive differential systems. A lot of work has been done in this direction to research impulsive differential equations, see a few viewpoints and the references therein [25–37]. They explored a generalized form of delayed Nicholson’s blowflies model with impulse

$$\begin{aligned} \textstyle\begin{cases} k' (t) = -a k(t) + \sum_{i=1}^{n} b_{i} k(t-t_{1}) e^{ - c_{i} k(t- t_{1})}, & t\neq \eta _{m}, \\ \Delta k( \eta _{m}) = d_{m} k(\eta _{m}), & t=\eta _{m}, \end{cases}\displaystyle \end{aligned}$$

to establish the existence of positive periodic solutions. In 2012, the authors considered a discrete Nicholson’s blowflies model involving a linear harvesting term, and with appropriate assumptions, sufficient conditions were established for the existence and exponential convergence of positive almost periodic solutions of the model [38]. Many authors have explored the discrete Nicholson’s blowflies model (for instance consider [39–42]). They derived the exponential extinction, exponential stability, exponential convergence of almost periodic and multiple periodic solutions. Furthermore, more discussions about periodic solution, global stability, and exponential stability of delayed Nicholson’s blowflies model could be found in references [43–56] by using of Schauder’s fixed point theorem, Krasnoselskii’s fixed point theorem, and Leggett–Williams fixed point theorem.

Here, we propose in this paper the following dual system of Nicholson’s blowflies model:

1 $$\begin{aligned} \textstyle\begin{cases} k_{1} (\nu +1) = k_{1}(\nu ) \exp ( - \frac{a_{11}(\nu )}{ b_{11}(\nu )+ k_{1}(\nu )} + \frac{a_{12}(\nu )}{b_{12}(\nu ) + k_{2}(\nu )} \cdot \frac{k_{2}(\nu )}{ k_{1}(\nu )} \\ \phantom{k_{1} (\nu +1) = }{} + \frac{c_{1}(\nu ) k_{1}( \nu - \tau _{1}(\nu ))}{ k_{1}(\nu ) } \exp ( - \delta _{1}(\nu ) k_{1}( \nu - \tau _{1}(\nu )) ) \\ \phantom{k_{1} (\nu +1) = }{} -\mathcal{H}_{1}(\nu ) \frac{k_{1}( \nu - \tau _{1}(\nu ))}{ k_{1}(\nu )} ), \\ k_{2}(\nu +1) = k_{2}(\nu ) \exp ( - \frac{a_{22}(\nu )}{ b_{22}(\nu ) + k_{2}(\nu )} + \frac{a_{21}(\nu )}{ b_{21}(\nu ) + k_{1}(\nu )} \cdot \frac{ k_{1}(\nu )}{ k_{2}(\nu )} \\ \phantom{k_{2}(\nu +1) =}{} + \frac{ c_{2}(\nu ) k_{2}( \nu - \tau _{2}(\nu ))}{ k_{2}(\nu )} \exp ( - \delta _{2}(\nu ) k_{2} (\nu - \tau _{2}(\nu )) ) \\ \phantom{k_{2}(\nu +1) =}{} - \mathcal{H}_{2}(\nu ) \frac{ k_{2}( \nu - \tau _{2}(\nu ))}{ k_{2}(\nu )} ), \end{cases}\displaystyle \end{aligned}$$

where $a_{ij}$, $b_{ij}$, $c_{i}$, $\delta _{i}$, $\mathcal{H}_{i}: \mathbb{R}\to (0, \infty )$ for $i, j = 1,2$, $k_{i}(t)$ ($i = 1,2$) denotes the population’s size at time *t* and periodic functions $\tau _{1}, \tau _{2}: \mathbb{R}\to [0, \infty )$ are continuous with period *p*. By using the technical idea of Gaines and Mawhin continuation theorem of coincidence degree theory in [57], we derive the sufficient conditions for the new result of existence of positive periodic solution to system (1). Finally, one numerical simulation example is provided to verify the main results.

We arrange the rest of the paper as follows: In Sect. 2, we recall some preliminaries of the basic tool. Section 3 is devoted to showing the main results, while an example illustrating the obtained results and an algorithm for the system are presented in Sect. 4.

## Preliminaries

Before exploring the existence of periodic solutions of the system, we give some denotations, which will be useful to prove the main result. Let $\mathscr{L}: \operatorname{Dom } \mathscr{L} \subset \mathcal{Y} \to \mathcal{Z}$ and $\mathscr{N}: \mathcal{Y} \to \mathcal{Z}$ be a linear mapping and a continuous mapping, respectively, where $\mathcal{Y}$ and $\mathcal{Z}$ are real Banach spaces. Let $\mathcal{Y}$ and $\mathcal{Z}$ be Banach spaces and $LB(\mathcal{Y}, \mathcal{Z})$ denote the set of bounded linear operators $\mathscr{T}$ from $\mathcal{Y}$ to $\mathcal{Z}$ with $\operatorname{Dom } (\mathscr{T})= \mathcal{Y}$. An operator $\mathscr{L} \in LB(\mathcal{Y}, \mathcal{Z})$ is called a Fredholm mapping of index zero if $\dim \ker \mathscr{L}, \operatorname{codim } \operatorname{Im } \mathscr{L}$ are finite and $\operatorname{Im } \mathscr{L} \subset \mathcal{Z}$ is closed. If $\mathscr{L}$ is a Fredholm mapping of index zero, then there exist continuous projectors $\mathscr{P}: \mathcal{Y} \to \mathcal{Y}$ and $\mathscr{Q}: \mathcal{Z} \to \mathcal{Z}$ such that

2 $$\begin{aligned} \operatorname{Im } \mathscr{P} = \ker \mathscr{L},\qquad \ker \mathscr{Q} = \operatorname{Im } \mathscr{L} = \operatorname{Im }( I - \mathscr{Q}). \end{aligned}$$

It follows that the restriction $\mathscr{L}|_{\mathscr{P}}$ of $\mathscr{L}$ to

$$\begin{aligned} \operatorname{Dom } \mathscr{L} \cap \ker \mathscr{P}: ( \mathscr{I} - \mathscr{P}) \mathcal{Y} \to \operatorname{Im } \mathscr{L} \end{aligned}$$

is invertible. Denote the inverse of $\mathscr{L}|_{\mathscr{ P}}$ by $\mathcal{K}$.

### Lemma 1

([57])

*Let* $\mathcal{O} \subset \mathcal{Y}$ *be an open bounded set*, $\mathscr{L}$ *be a Fredholm mapping of index zero*, *and* $\mathscr{N}$ *be* $\mathscr{L}$-*compact on* $\overline{\mathcal{O}}$. *Assume*

(I) $\mathscr{L} (z) \neq \eta \mathscr{N} (z)$ *for all* $\eta \in (0,1)$, $z \in \partial \mathcal{O} \cap \operatorname{Dom } \mathscr{L}$;
(II) $\mathscr{Q} (\mathscr{N} (z)) \neq 0$ *for each* $z \in \partial \mathcal{O} \cap \ker \mathscr{L}$;
(III) $\deg ( J\mathscr{Q} \mathscr{N}, \mathcal{O} \cap \ker \mathscr{L}, 0 ) \neq 0$.

*Then* $\mathscr{L} (z) = \mathscr{N} (z)$ *has at least one solution in* $\overline{ \mathcal{O}} \cap \operatorname{Dom } \mathscr{L}$.

### Lemma 2

([58])

*Suppose that* $k: \mathbb{Z} \to \mathbb{R}$ *is a* *p*-*periodic function such that* $k( \nu + p)= k(\nu )$. *Then*, *for any fixed* $\nu _{1}$, $\nu _{2}$ *belonging to*

$$\begin{aligned} I_{p}= \{0,1,\dots, p-1 \} \end{aligned}$$

*and any* $\nu \in \mathbb{Z}$, *one has*

$$\begin{aligned} k(\nu _{2})-\sum_{\nu =0}^{p-1} \bigl\vert k(\nu +1) - k(\nu ) \bigr\vert \leq k(\nu ) \leq k(\nu _{1}) + \sum_{\nu =0}^{p-1} \bigl\vert k ( \nu + 1) - k(\nu ) \bigr\vert . \end{aligned}$$

For convenience, we shall introduce the following notations:

$$\begin{aligned} \overline{g} = \frac{1}{p} \sum_{ \nu =0}^{ p-1}g( \nu ),\qquad g^{\ast }=\max_{\nu \in I_{p}} g(\nu ),\qquad g_{\ast }= \min_{\nu \in I_{p}} g(\nu ). \end{aligned}$$

## Main results

### Theorem 3

*For system *(1), *we assume that*:

1. $a_{ij}(\nu )$, $b_{ij}(\nu )$, $c_{i}(\nu )$, $\delta _{1}(\nu )$, *and* $\mathcal{H}_{i}(\nu )$ *are continuous positive periodic functions with period* $p > 0$ *and* $\tau _{i}(\nu )$ *is a nonnegative continuous function with*
  $$\begin{aligned} \tau _{i}(\nu ) = \tau _{i} ( \nu + p), \end{aligned}$$
  *here* $i, j = 1,2$.
2. $\frac{a_{11}^{\ast }}{ b_{11}}_{ \ast } + \mathcal{H}_{1}^{\ast }>0$ *and* $\frac{a_{22}^{\ast }}{b_{22}}_{\ast } + \mathcal{H}_{2}^{\ast }>0$.
3. ${\mathcal{H}_{1}}_{\ast } > \frac{a_{12}^{ \ast }}{{b_{12}}_{\ast }}$ *and* ${\mathcal{H}_{2}}_{ \ast } > \frac{a_{21}^{ \ast }}{{b_{21}}_{\ast }}$,

*here*

$$\begin{aligned} a_{ij}^{\ast } = \max_{\nu \in I_{p}} a_{ij}( \nu ),\qquad {b_{ij}}_{ \ast }= \min_{\nu \in I_{p}} b_{ij}(\nu ),\quad (i, j =1,2) \end{aligned}$$

*and*

3 $$\begin{aligned} \mathcal{H}_{i}^{\ast } = \max _{\nu \in I_{p}} \mathcal{H}_{i}( \nu ),\qquad {\mathcal{H}_{i}}_{\ast } = \min_{\nu \in I_{p}} \mathcal{H}_{i}(\nu ). \end{aligned}$$

*Then there exists at least one positive periodic solution of system *(1).

### Proof

By the biological meaning, we only focus on the positive periodic solutions to system (1). Let the transformation be $k_{1}(\nu ) = \exp (\varrho _{1}(\nu ))$ and $k_{2}(\nu ) = \exp (\varrho _{2}(\nu ))$. Then system (1) becomes

4 $$\begin{aligned} \textstyle\begin{cases} \varrho _{1}(\nu + 1) - \varrho _{1}(\nu ) = \theta _{1}(\nu ), \\ \varrho _{2}(\nu + 1) - \varrho _{2}(\nu ) = \theta _{2}(\nu ), \end{cases}\displaystyle \end{aligned}$$

where

5 $$\begin{aligned} \theta _{1} (\nu ) ={}& {-} \frac{ a_{11}( \nu )}{ b_{11}(\nu ) + \exp (\varrho _{1}(\nu ) )} \\ &{} + \frac{ a_{12}(\nu )}{ b_{12}(\nu ) + \exp (\varrho _{2}(\nu )) } \exp \bigl( \varrho _{2}(\nu ) - \varrho _{1}(\nu ) \bigr) \\ &{} - \mathcal{H}_{1}(\nu ) \exp \bigl(\varrho _{1}\bigl( \nu - \tau _{1}( \nu )\bigr) - \varrho _{1}(\nu ) \bigr) \\ &{} + c_{1}(\nu ) \exp \bigl( \varrho _{1} \bigl( \nu - \tau _{1}(\nu )\bigr)- \varrho _{1}(\nu ) - \delta _{1}(\nu ) \exp \bigl( \varrho _{1}\bigl( \nu - \tau _{1}(\nu ) \bigr)\bigr) \bigr), \\ \theta _{2}(\nu ) ={}&{ -} \frac{ a_{22}(\nu )}{ b_{22}(\nu ) + \exp ( \varrho _{2}(\nu )) } \\ &{} + \frac{a_{21}(\nu )}{ b_{21}(\nu ) + \exp (\varrho _{1}(\nu )) } \exp \bigl( \varrho _{1}(\nu ) - \varrho _{2}(\nu ) \bigr) \\ & {}- \mathcal{H}_{2}(\nu ) \exp \bigl( \varrho _{2}\bigl( \nu -\tau _{2}( \nu )\bigr)-\varrho _{2}(\nu ) \bigr) \\ & {}+ c_{2}(\nu ) \exp \bigl(\varrho _{2}\bigl( \nu - \tau _{2}(\nu )\bigr) - \varrho _{2}(\nu ) - \delta _{2}(\nu ) \exp \bigl(\varrho _{2}\bigl(\nu -\tau _{2}( \nu )\bigr)\bigr) \bigr). \end{aligned}$$

Since (4) has a *p*-periodic solution $(\varrho _{1}(\nu ), \varrho _{2}(\nu ))^{T}$, it is easy to see that

$$\begin{aligned} \bigl( k_{1}(\nu ), k_{2}(\nu ) \bigr)^{T} = \bigl( \exp \bigl( \varrho _{1}(\nu )\bigr), \exp \bigl(\varrho _{2}(l)\bigr) \bigr)^{T} \end{aligned}$$

is a positive *p*-periodic solution of (1). Next, it needs to show that (4) has a *p*-periodic solution. Define

$$\begin{aligned} j_{2} = \bigl\{ z = \bigl(z(\nu )\bigr): z(\nu ) \in \mathbb{R}^{2}, \nu \in \mathbb{Z} \bigr\} . \end{aligned}$$

For $r = (r_{1}, r_{2})^{T} \in \mathbb{R}^{2}$, define $|r| = \max \{|r_{1}|, |r_{2}|\}$. Let $j^{p} \subset j_{2}$ denote the subspace of all *p*-periodic sequences equipped with the usual supremum norm $\|\cdot\|$, i.e.,

$$\begin{aligned} \Vert z \Vert = \max_{\nu \in I_{p}} \bigl\vert z(\nu ) \bigr\vert , \end{aligned}$$

for any

$$\begin{aligned} z = \bigl\{ z(\nu ): \nu \in \mathbb{Z} \bigr\} \in j^{p}. \end{aligned}$$

It is obvious that $j^{p}$ is a finite dimensional Banach space. Let

$$\begin{aligned} j_{0}^{p} = \Biggl\{ z = \bigl(z(\nu )\bigr) \in j^{p}: \sum_{l=0}^{p-1} z(\nu ) = 0 \Biggr\} \end{aligned}$$

and

$$\begin{aligned} j_{c}^{p} = \bigl\{ z = \bigl(z(\nu )\bigr) \in j^{p}: z(\nu ) = h \in \mathbb{R}^{2}, \nu \in \mathbb{Z} \bigr\} . \end{aligned}$$

Then it follows that $j_{0}^{p}$ and $j_{c}^{p}$ are both closed linear subspaces of $j^{p}$ and $j^{p} = j_{c}^{p} \oplus j_{0}^{p}$, $\dim j_{c}^{p} = 2$. We take

$$\begin{aligned} j^{p} = \mathcal{Z} = \bigl\{ z(\nu ) = \bigl( \varrho _{1}(\nu ), \varrho _{2}( \nu )\bigr)^{T} \in \mathbb{R}^{2}: \varrho _{1}(\nu + p) = \varrho _{1}( \nu ), \varrho _{2}(\nu + p) = \varrho _{2} (\nu ) \bigr\} \end{aligned}$$

and

$$\begin{aligned} \Vert z \Vert = \bigl\Vert \bigl(\varrho _{1}(\nu ), \varrho _{2}(\nu )\bigr)^{T} \bigr\Vert = \max _{ \nu \in I_{p}} \bigl\vert \varrho _{1} (\nu ) \bigr\vert + \max_{ \nu \in I_{p} } \bigl\vert \varrho _{2}(\nu ) \bigr\vert . \end{aligned}$$

Then $\mathcal{Z}$ is a Banach space with norm $\|\cdot\|$. Now, we define $\mathscr{L}: \operatorname{Dom }(\mathscr{L}) \subset \mathcal{Z} \to \mathcal{Z}$ and $\mathscr{N}: \mathcal{Z} \to \mathcal{Z}$ by

$$\begin{aligned} \mathscr{L} (\varrho _{1}, \varrho _{2} ) = \begin{pmatrix} \varrho _{1}(\nu + 1) - \varrho _{1} (\nu ) \\ \varrho _{2} ( \nu + 1) - \varrho _{2}(\nu ) \end{pmatrix} \end{aligned}$$

and

$$\begin{aligned} \mathscr{N} ( \varrho _{1}, \varrho _{2} )= \begin{pmatrix} \theta _{1}(\nu ) \\ \theta _{2}(\nu ) \end{pmatrix} \end{aligned}$$

for any $(\varrho _{1}, \varrho _{2})^{T} \in \mathcal{Z}$ and $\nu \in \mathbb{Z}$, here $\theta _{1}(\nu )$ and $\theta _{2}(\nu )$ are defined by Eq. (5). It is trivial to get the argument $\mathscr{L}$ is a bounded linear operator, $\ker \mathscr{L}= j_{c}^{p}$, $\operatorname{Im } \mathscr{L} = j_{0}^{p}$, and $\operatorname{Im } \mathscr{L} \subset \mathcal{Z}$ is closed. Therefore, $\dim \ker \mathscr{L} = \operatorname{codim } \operatorname{Im } \mathscr{L}= 2$. Indeed, $\mathscr{L} $ is a Fredholm mapping of index zero. At present, we set the continuous projectors $\mathscr{P}: \mathcal{Z}\to \mathcal{Z}$ and $\mathscr{Q}: \mathcal{Z} \to \mathcal{Z}$ defined by

$$\begin{aligned} \mathscr{P} (\varrho _{1}, \varrho _{2} )= \mathscr{Q} ( \varrho _{1}, \varrho _{2} ) = \begin{pmatrix} \frac{1}{p} [\sum_{\nu =0}^{p-1}\varrho _{1}(\nu ) ] \\ \frac{1}{p} [\sum_{\nu =0}^{p-1}\varrho _{2}(\nu ) ] \end{pmatrix}, \end{aligned}$$

such that Eq. (2) holds. Furthermore, $\mathcal{K}$ denotes the inverse of $\mathscr{L} |_{ \operatorname{Dom } \mathscr{K} \cap \ker \mathscr{P}}$,

$$\begin{aligned} \mathcal{K} ( \varrho _{1}, \varrho _{2} )= \begin{pmatrix} \sum_{\nu =0}^{ p-1} \varrho _{1}(\nu ) - \frac{1}{p} [ \sum_{\nu =0}^{ p-1}(p-\nu ) \varrho _{1}(\nu ) ] \\ \sum_{\nu =0}^{ p-1} \varrho _{2}(\nu ) - \frac{1}{p} [ \sum_{\nu =0}^{p-1} (p-\nu )\varrho _{2}(\nu ) ] \end{pmatrix}, \end{aligned}$$

$\mathscr{Q} \mathscr{N}: \mathcal{Z} \to \mathcal{Z}$ and $\mathcal{K}(I - \mathscr{Q}) \mathscr{N}: \mathcal{Z} \to \mathcal{Z}$ are defined by

$$\begin{aligned} &\mathscr{Q}\mathscr{N} (\varrho _{1}, \varrho _{2} ) = \begin{pmatrix} \frac{1}{p} \sum_{\nu =0}^{p-1} \theta _{1}(\nu ) \\ \frac{1}{p} \sum_{\nu =0}^{p-1} \theta _{2}(\nu ) \end{pmatrix}, \\ &\mathcal{K} (I-\mathscr{Q}) \mathscr{N} ( \varrho _{1}, \varrho _{2} ) = \begin{pmatrix} \sum_{\nu =0}^{ p-1} \theta _{1}(\nu ) \\ \sum_{\nu =0}^{p-1} \theta _{2}(\nu ) \end{pmatrix} - \begin{pmatrix} \frac{1}{p} \sum_{\nu =0}^{ p-1}(p-\nu ) \theta _{1}(\nu ) \\ \frac{1}{p}\sum_{\nu =0}^{p-1}(p-\nu ) \theta _{2}(\nu ) \end{pmatrix} \\ &\phantom{\mathcal{K} (I-\mathscr{Q}) \mathscr{N} ( \varrho _{1}, \varrho _{2} )=}{} - \begin{pmatrix} ( \frac{\nu }{p} - \frac{p+1}{2p} ) \sum_{\nu =0}^{p-1} \theta _{1}(\nu ) \\ ( \frac{\nu }{p} - \frac{p+1}{2p} ) \sum_{\nu =0}^{p-1} \theta _{2}(\nu ) \end{pmatrix}. \end{aligned}$$

This implies that functions $\mathscr{Q}\mathscr{N}$ and $\mathcal{K}(I - \mathscr{Q}) \mathscr{N}$ are all continuous. On the other hand, $\mathcal{Z}$ is a finite dimensional Banach space, and so the Arzelà–Ascoli theorem implies that

$$\begin{aligned} \overline{ \mathcal{K}( I -\mathscr{Q}) \mathscr{N} (\overline{\mathcal{O}})} \end{aligned}$$

is compact for any open bounded set $\mathcal{O} \subset \mathcal{Z}$. Moreover, $\mathscr{Q}\mathscr{N}( \overline{\mathcal{O}} )$ is bounded. Therefore, $\mathscr{N}$ is $\mathscr{L}$-compact on $\overline{ \mathcal{O}}$ for any open bounded set $\mathcal{O} \subset \mathcal{Z}$. The isomorphism $J: \operatorname{Im } \mathscr{Q} \to \ker \mathscr{L}$ is an identity mapping such that $\ker \mathscr{L} =\operatorname{Im } \mathscr{Q}$. In the following, we consider the operator equation $\mathscr{L} \varrho = \eta \mathscr{N} \varrho $ for $\eta \in (0,1)$, that is,

6 $$\begin{aligned} \textstyle\begin{cases} \varrho _{1}(\nu +1) - \varrho _{1}(\nu ) = \eta \theta _{1}(\nu ), \\ \varrho _{2}(\nu +1)-\varrho _{2}(\nu ) = \eta \theta _{2}(\nu ). \end{cases}\displaystyle \end{aligned}$$

Suppose that $(\varrho _{1}(\nu ), \varrho _{2}(\nu ))^{T} \in \mathcal{Z}$ is a solution of (6) for certain $\eta \in (0,1)$, and summing from 0 to $p - 1$ on both sides, we get

7 $$\begin{aligned} \textstyle\begin{cases} \sum_{\nu =0}^{p-1} \theta _{1}(\nu )=0, \\ \sum_{\nu =0}^{p-1} \theta _{2}(\nu ) =0. \end{cases}\displaystyle \end{aligned}$$

Combining the first equation of system (6) and the first equation of system (7), we have

8 $$\begin{aligned} \sum_{\nu =0}^{p-1} \bigl\vert \varrho _{1}(\nu +1) - \varrho _{1}( \nu ) \bigr\vert < M_{1}, \end{aligned}$$

here

$$\begin{aligned} M_{1} = 2 \sum_{\nu =0}^{p-1} \frac{a_{11}(\nu )}{b_{11}(\nu )}. \end{aligned}$$

From the second equation of system (6) and the second equation of system (7), we have

9 $$\begin{aligned} \sum_{\nu =0}^{p-1} \bigl\vert \varrho _{2}(\nu +1) - \varrho _{2}( \nu ) \bigr\vert < M_{2}, \end{aligned}$$

here

$$\begin{aligned} M_{2} = 2 \sum_{\nu =0}^{p-1} \frac{a_{22}( \nu )}{ b_{22}(\nu )}. \end{aligned}$$

Multiplying the first equation of system (7) by $\exp (\varrho _{1}(l))$, we obtain

10 $$\begin{aligned} & \sum_{\nu =0}^{p-1} \frac{ a_{11}(\nu )}{ b_{11}(\nu ) + \exp (\varrho _{1}(\nu )) } \exp \bigl(\varrho _{1} (\nu )\bigr) + \sum _{\nu =0}^{p-1} \mathcal{H}_{1}( \nu ) \exp \bigl(\varrho _{1}\bigl( \nu -\tau _{1}(\nu )\bigr)\bigr) \\ &\quad = \sum_{\nu =0}^{p-1} \frac{ a_{12}(\nu )}{ b_{12}(\nu ) + \exp (\varrho _{2}(\nu )) } \exp \bigl(\varrho _{2} (\nu )\bigr) \\ &\qquad{} + \sum_{\nu =0}^{p-1} c_{1}(\nu ) \exp \bigl( \varrho _{1}\bigl( \nu -\tau _{1}(\nu )\bigr) - \delta _{1}(\nu ) e^{\varrho _{1}( \nu -\tau _{1}(\nu ))} \bigr). \end{aligned}$$

Notice that

11 $$\begin{aligned} \begin{aligned}& \sum_{\nu =0}^{p-1} \exp \bigl(\varrho _{1}\bigl(\nu - \tau _{1}(\nu )\bigr) \bigr) = \sum_{\nu =0}^{p-1} \exp \bigl(\varrho _{1} ( \nu )\bigr), \\ &\sum_{\nu =0}^{p-1} \exp \bigl(\varrho _{2} \bigl( \nu - \tau _{2}( \nu )\bigr)\bigr) = \sum _{\nu =0}^{p-1} \exp \bigl( \varrho _{2}(\nu )\bigr) \end{aligned} \end{aligned}$$

and

12 $$\begin{aligned} \sup_{\varrho \geq 0} \varrho \exp (-\varrho ) = \frac{1}{\exp (1)}. \end{aligned}$$

Since $(\varrho _{1}(\nu ), \varrho _{2}(\nu ))^{T}\in \mathcal{Z}$, there exist $\alpha _{i}$, $\beta _{i}\in I_{p}$ such that

13 $$\begin{aligned} \varrho _{i}(\alpha _{i}) = \max _{\nu \in I_{p}} \varrho _{i}( \nu ),\qquad \varrho _{i}( \beta _{i}) = \min_{\nu \in I_{p}} \varrho _{i}(\nu ), \end{aligned}$$

with $i=1,2$. Substituting (11), (12), and (13) in (10), we get

$$\begin{aligned} &\sum_{\nu =0}^{p-1} \frac{a_{11}(\nu )}{ b_{11}(\nu ) + \exp (\varrho _{1}(\nu ))} \exp \bigl( \varrho _{1} (\nu )\bigr) + \sum_{\nu =0}^{p-1} \mathcal{H}_{1}( \nu ) \exp \bigl(\varrho _{1}(\nu )\bigr) \\ &\quad = \sum_{\nu =0}^{p-1} \frac{a_{12}(\nu )}{b_{12}(\nu ) + \exp (\varrho _{2}(\nu ))} \exp \bigl( \varrho _{2}(\nu )\bigr) \\ &\qquad{} + \sum_{\nu =0}^{p-1} \frac{c_{1}(\nu )}{ \delta _{1}(\nu )} \delta _{1}(\nu ) \exp \bigl( \varrho _{1}\bigl(\nu -\tau _{1}(\nu )\bigr) - \delta _{1}(\nu ) \exp \bigl(\varrho _{1}\bigl( \nu -\tau _{1}(\nu )\bigr)\bigr) \bigr), \\ &\biggl[ \frac{ {a_{11}}^{\ast }}{ {b_{11}}_{\ast }} + {\mathcal{H}_{1}}^{ \ast } \biggr] \sum_{\nu =0}^{p-1} \exp \bigl(\varrho _{1}(\nu )\bigr) > \sum_{ \nu =0}^{p-1} \frac{c_{1}(\nu )}{ \delta _{1}(\nu )\exp (1) }, \\ &\biggl[ \frac{ {a_{11}}^{\ast }}{{ b_{11}}_{\ast }} + {\mathcal{H}_{1}}^{ \ast } \biggr] \exp \bigl(\varrho _{1}(\alpha _{1})\bigr) > \frac{{c_{1}}_{\ast }}{{ \delta _{1}^{\ast }}\exp (1) } \end{aligned}$$

and

14 $$\begin{aligned} \varrho _{1}(\alpha _{1}) > \log \biggl[ \frac{{c_{1}}_{\ast }{b_{11}}_{\ast }}{{\delta _{1}^{ \ast }} \exp (1)({a_{11}}^{ \ast } + {b_{11}}_{\ast }{ \mathcal{H}_{1}}^{\ast })} \biggr]. \end{aligned}$$

Multiplying the second equation of system (7) by $\exp ( \varrho _{2}(\nu ) )$, we obtain

15 $$\begin{aligned} & \sum_{\nu =0}^{p-1} \frac{a_{22}(\nu )}{ b_{22}(\nu ) + \exp (\varrho _{2}(\nu )) } \exp \bigl( \varrho _{2}(\nu )\bigr) + \sum _{\nu =0}^{p-1} \mathcal{H}_{2}(\nu ) \exp \bigl(\varrho _{2}\bigl(\nu -\tau _{2}(\nu )\bigr)\bigr) \\ &\quad =\sum_{\nu =0}^{p-1} \frac{a_{21}(\nu )}{b_{21}(\nu ) + \exp (\varrho _{1}(\nu )) } \exp \bigl( \varrho _{1}(\nu )\bigr) \\ &\qquad{} + \sum_{\nu =0}^{p-1} c_{2}(\nu ) \exp \bigl(\varrho _{2}\bigl( \nu -\tau _{2}(\nu )\bigr)- \delta _{2}(\nu )\exp \bigl( \varrho _{2}\bigl(\nu -\tau _{2}( \nu )\bigr)\bigr) \bigr). \end{aligned}$$

Substituting Eqs. (11), (12), and (13) in (15), we get

$$\begin{aligned} &\sum_{\nu =0}^{p-1} \frac{a_{22}(\nu )}{ b_{22}(\nu ) + \exp (\varrho _{2}(\nu )) } \exp \bigl( \varrho _{2}(\nu )\bigr) + \sum_{\nu =0}^{p-1} \mathcal{H}_{2}(\nu ) \exp \bigl( \varrho _{2}(\nu ) \bigr) \\ &\quad = \sum_{\nu =0}^{p-1} \frac{ a_{21}(\nu )}{ b_{21}(\nu ) + \exp (\varrho _{1}(\nu )) } \exp \bigl(\varrho _{1}(\nu )\bigr) \\ & \qquad{}+ \sum_{\nu =0}^{p-1} \frac{c_{2}(\nu )}{ \delta _{2}(\nu )} \delta _{2}(\nu ) \exp \bigl(\varrho _{2}\bigl( \nu - \tau _{2}(\nu )\bigr)- \delta _{2}(\nu ) \exp \bigl(\varrho _{2}\bigl(\nu -\tau _{2}(\nu )\bigr)\bigr) \bigr), \\ &\biggl[ \frac{{a_{22}}^{ \ast }}{{ b_{22}}_{\ast }} + {\mathcal{H}_{2}}^{ \ast } \biggr] \sum_{\nu =0}^{p-1} \exp \bigl(\varrho _{2}(\nu )\bigr) > \sum_{ \nu =0}^{p-1} \frac{c_{2}(\nu )}{ \delta _{2}(\nu )\exp (1)}, \\ &\biggl[ \frac{{a_{22}}^{ \ast }}{{b_{22}}_{\ast }} + {\mathcal{H}_{2}}^{ \ast } \biggr] \exp \bigl(\varrho _{2}(\alpha _{2})\bigr) > \frac{{c_{2}}_{\ast }}{{ \delta _{2}^{\ast }}\exp (1)} \end{aligned}$$

and

16 $$\begin{aligned} \varrho _{2}(\eta _{2}) > \log \biggl[ \frac{{c_{2}}_{\ast }{b_{22}}_{\ast }}{{\delta _{2}^{ \ast }} \exp (1)( { a_{22}}^{\ast } + { b_{22}}_{ \ast }{\mathcal{H}_{2}}^{\ast })} \biggr]. \end{aligned}$$

From Eqs. (10) and (11), we obtain

17 $$\begin{aligned} \sum_{\nu =0}^{p-1} \mathcal{H}_{1}(\nu ) \exp \bigl( \varrho _{1}(\nu )\bigr) < {}&\sum_{\nu =0}^{p-1} \frac{a_{12}(\nu )}{ b_{12}(\nu ) + \exp (\varrho _{2}(\nu ))} \exp \bigl( \varrho _{2}(\nu )\bigr) \\ &{} + \sum_{\nu =0}^{p-1} c_{1}(\nu ) \exp \bigl(\varrho _{1}\bigl( \nu -\tau _{1}(\nu )\bigr) - \delta _{1}(\nu ) \\ &{} \times \exp \bigl(\varrho _{1}\bigl(\nu -\tau _{1}(\nu ) \bigr)\bigr) \bigr). \end{aligned}$$

If

$$\begin{aligned} \sum_{\nu =0}^{p-1} \exp \bigl(\varrho _{2}(\nu )\bigr) \leq \sum_{\nu =0}^{p-1} \exp \bigl(\varrho _{1}(\nu )\bigr), \end{aligned}$$

and (12), then it follows from Eqs. (13) and (17) that

$$\begin{aligned} &\sum_{\nu =0}^{p-1} \mathcal{H}_{1}( \nu ) \exp \bigl(\varrho _{1}( \nu )\bigr) < \sum _{\nu =0}^{p-1} \frac{a_{12}(\nu )}{ b_{12}(\nu ) + \exp (\varrho _{2}(\nu )) } \exp \bigl(\varrho _{1}(\nu )\bigr) \\ &\phantom{\sum_{\nu =0}^{p-1} \mathcal{H}_{1}( \nu ) \exp \bigl(\varrho _{1}( \nu )\bigr) < }{} + \sum_{\nu =0}^{p-1} c_{1}(\nu ) \exp \bigl( \varrho _{1}\bigl(\nu - \tau _{1}(\nu )\bigr) - \delta _{1}(\nu ) \\ &\phantom{\sum_{\nu =0}^{p-1} \mathcal{H}_{1}( \nu ) \exp \bigl(\varrho _{1}( \nu )\bigr) < }{} \times \exp \bigl(\varrho _{1}\bigl(\nu -\tau _{1}(\nu )\bigr)\bigr) \bigr), \\ &\biggl[ {H_{1}}_{\ast } - \frac{{a_{12}}^{ \ast }}{{ b_{12}}_{ \ast }} \biggr] \sum _{\nu =0}^{p-1} \exp \bigl(\varrho _{2}(\nu )\bigr) \leq \biggl[{ \mathcal{H}_{1}}_{\ast } - \frac{{a_{12}}^{\ast }}{{ b_{12}}_{ \ast }} \biggr] \sum_{\nu =0}^{p-1} \exp \bigl(\varrho _{1}(\nu )\bigr) \\ &\phantom{\biggl[ {H_{1}}_{\ast } - \frac{{a_{12}}^{ \ast }}{{ b_{12}}_{ \ast }} \biggr] \sum _{\nu =0}^{p-1} \exp \bigl(\varrho _{2}(\nu )\bigr)} < \sum_{\nu =0}^{p-1} \frac{c_{1}(\nu )}{\delta _{1}( \nu )\exp (1)} \end{aligned}$$

and

18 $$\begin{aligned} \varrho _{2}( \beta _{2}) < \log \biggl[ \frac{{c_{1}}^{\ast }{ b_{12}}_{\ast }}{{ \delta _{1}}_{\ast } \exp (1)({{b_{12}}_{\ast }{\mathcal{H}_{1}}_{\ast } - a_{12}}^{\ast })} \biggr]. \end{aligned}$$

From (11) and (15) we obtain

19 $$\begin{aligned} \sum_{\nu =0}^{p-1} \mathcal{H}_{2}(\nu ) \exp \bigl(\varrho _{2}(\nu )\bigr) < {}& \sum_{\nu =0}^{p-1} \frac{a_{21}(\nu )}{b_{21}(\nu ) + \exp (\varrho _{1}(\nu )) } \exp \bigl( \varrho _{1}(\nu )\bigr) \\ &{} + \sum_{\nu =0}^{p-1} c_{2}(\nu ) \exp \bigl(\varrho _{2}\bigl( \nu - \tau _{2}(\nu )\bigr) - \delta _{2}(\nu ) \exp \bigl(\varrho _{2}\bigl(\nu -\tau _{2}(\nu ) \bigr)\bigr) \bigr). \end{aligned}$$

If

$$\begin{aligned} \sum_{\nu =0}^{p-1} \exp \bigl(\varrho _{1}(\nu )\bigr) \leq \sum_{\nu =0}^{p-1} \exp \bigl(\varrho _{2}(\nu )\bigr) \end{aligned}$$

and (12), then from (13) and (19) we obtain

$$\begin{aligned} &\sum_{\nu =0}^{p-1} \mathcal{H}_{2}( \nu ) \exp \bigl( \varrho _{2}( \nu )\bigr) < \sum _{\nu =0}^{p-1} \frac{a_{21}(\nu )}{ b_{21}(\nu ) + \exp (\varrho _{1}(\nu ) } \exp \bigl( \varrho _{2}(\nu )\bigr) \\ &\phantom{\sum_{\nu =0}^{p-1} \mathcal{H}_{2}( \nu ) \exp \bigl( \varrho _{2}( \nu )\bigr) < }{} + \sum_{\nu =0}^{p -1} c_{2}(\nu ) \exp \bigl( \varrho _{2}\bigl( \nu -\tau _{2}(\nu )\bigr) - \delta _{2}(\nu ) \\ &\phantom{\sum_{\nu =0}^{p-1} \mathcal{H}_{2}( \nu ) \exp \bigl( \varrho _{2}( \nu )\bigr) < }{} \times \exp \bigl(\varrho _{2}\bigl(\nu -\tau _{2}(\nu ) \bigr)\bigr) \bigr), \\ &\biggl[ {\mathcal{H}_{2}}_{\ast } - \frac{{a_{21}}^{\ast }}{{ b_{21}}_{\ast }} \biggr] \sum_{\nu =0}^{p-1} \exp \bigl(\varrho _{1}(\nu )\bigr) \leq \biggl[{\mathcal{H}_{2}}_{\ast } - \frac{{a_{21}}^{\ast }}{{ b_{21}}_{ \ast }} \biggr] \sum_{\nu =0}^{p-1} \exp \bigl(\varrho _{2}(\nu )\bigr) \\ &\phantom{\biggl[ {\mathcal{H}_{2}}_{\ast } - \frac{{a_{21}}^{\ast }}{{ b_{21}}_{\ast }} \biggr] \sum_{\nu =0}^{p-1} \exp \bigl(\varrho _{1}(\nu )\bigr)} < \sum_{\nu =0}^{p-1} \frac{c_{2}(\nu )}{\delta _{2}(\nu )\exp (1)}, \end{aligned}$$

and

20 $$\begin{aligned} \varrho _{1}( \beta _{1}) < \log \biggl[ \frac{{c_{2}}^{\ast }{ b_{21}}_{ \ast }}{{ \delta _{2}}_{ \ast } \exp (1) ({{b_{21}}_{ \ast }{ \mathcal{H}_{2}}_{\ast } - a_{21}}^{\ast })} \biggr]. \end{aligned}$$

From Lemma (2), (8), (9), (14), and (16), we obtain

21 $$\begin{aligned} \textstyle\begin{cases} \varrho _{1}(\nu ) \geq \varrho _{1}(\alpha _{1}) - \sum_{ \nu =0}^{p-1} \vert \varrho _{1}(\nu +1) - \varrho _{1}(\nu ) \vert \\ \phantom{\varrho _{1}(\nu )}> \log [ \frac{{c_{1}}_{\ast }{ b_{11}}_{\ast }}{{ \delta _{1}^{ \ast }} \exp (1) ({a_{11}}^{\ast } + {b_{11}}_{\ast }{\mathcal{H}_{1}}^{\ast })} ] - M_{1}, \\ \varrho _{2}(\nu ) \geq \varrho _{1}(\alpha _{2}) - \sum_{ \nu =0}^{p-1} \vert \varrho _{2}(\nu +1) - \varrho _{2}(\nu ) \vert \\ \phantom{\varrho _{2}(\nu ) } > \log [ \frac{{c_{2}}_{\ast }{b_{22}}_{\ast }}{{ \delta _{2}^{ \ast }} \exp (1)({a_{22}}^{ \ast } + {b_{22}}_{\ast }{ \mathcal{H}_{2}}^{ \ast })} ] - M_{2}.\end{cases}\displaystyle \end{aligned}$$

From Lemma 2, equations (8), (9), (18), and (20), we get

22 $$\begin{aligned} \textstyle\begin{cases} \varrho _{1}(\nu ) \leq \varrho _{1}( \beta _{1}) + \sum_{ \nu =0}^{p-1} \vert \varrho _{1}(\nu +1) - \varrho _{1}(\nu ) \vert \\ \phantom{\varrho _{1}(\nu )} < \log [ \frac{{c_{2}}^{ \ast }{ b_{21}}_{ \ast }}{{\delta _{2}}_{ \ast } \exp (1) ({{b_{21}}_{ \ast }{ \mathcal{H}_{2}}_{ \ast } - a_{21} }^{ \ast })} ] + M_{1}, \\ \varrho _{2}(\nu ) \leq \varrho _{2}( \beta _{2}) + \sum_{ \nu =0}^{p-1} \vert \varrho _{2}(\nu +1) - \varrho _{2}(\nu ) \vert \\ \phantom{\varrho _{1}(\nu )} < \log [ \frac{{c_{1}}^{ \ast }{ b_{12}}_{ \ast }}{{ \delta _{1}}_{ \ast }\exp (1) ({{b_{12}}_{ \ast }{ \mathcal{H}_{1}}_{\ast } - a_{12}}^{ \ast })} ] + M_{2}. \end{cases}\displaystyle \end{aligned}$$

From the first equations of (21) and (22), we have $\max_{\nu \in I_{p}}|\varrho _{1}(\nu )| < S_{1}$, where

23 $$\begin{aligned} S_{1} ={}& \max \biggl\{ \biggl\vert \log \biggl[ \frac{ {c_{1}}_{\ast }{ b_{11}}_{ \ast }}{{ \delta _{1}^{ \ast }} \exp (1) ( {a_{11}}^{ \ast } + {b_{11}}_{\ast }{ \mathcal{H}_{1}}^{\ast })} \biggr] \biggr\vert + M_{1}, \\ & \biggl\vert \log \biggl[ \frac{{c_{2}}^{\ast }{ b_{21}}_{\ast }}{{ \delta _{2}}_{\ast } \exp (1)({{b_{21}}_{\ast }{ \mathcal{H}_{2}}_{\ast } - a_{21}}^{ \ast })} \biggr] \biggr\vert + M_{1} \biggr\} . \end{aligned}$$

By a similar argument, the second equations of (21) and (22) imply that $\max_{\nu \in I_{p}} |\varrho _{2}(\nu )| < S_{2}$, where

24 $$\begin{aligned} S_{2} ={}& \max \biggl\{ \biggl\vert \log \biggl[ \frac{{c_{2}}_{\ast }{ b_{22}}_{\ast }}{{ \delta _{2}^{ \ast }} \exp (1) ({ a_{22}}^{ \ast } + { b_{22}}_{ \ast }{ \mathcal{H}_{2}}^{\ast })} \biggr] \biggr\vert + M_{2}, \\ & \biggl\vert \log \biggl[ \frac{{c_{1}}^{ \ast }{b_{12}}_{ \ast }}{{ \delta _{1}}_{ \ast } \exp (1)({{b_{12}}_{ \ast }{ \mathcal{H}_{1}}_{\ast } - a_{12}}^{ \ast })} \biggr] \biggr\vert + M_{2} \biggr\} . \end{aligned}$$

Clearly, $S_{1}$ and $S_{2}$ are independent of *η*. Denote $S=S_{1}+S_{2} + S_{0}$, where $S_{0}$ is sufficiently large such that each solution $(\varrho _{1}, \varrho _{2})^{T}$ of the system of algebraic equations

$$\begin{aligned} \textstyle\begin{cases} {-} \frac{ \overline{a_{11}}}{ \overline{b_{11}} + \exp (\varrho _{1}) } + \frac{ \overline{ a_{12}}}{ \overline{ b_{12}} + \exp (\varrho _{2}) } \exp (\varrho _{2} - \varrho _{1} ) - \overline{ \mathcal{H}_{1}} \\ \quad{} + \overline{ c_{1}} \exp (-\overline{\delta _{1}} \exp (\varrho _{1}) )=0, \\ {-} \frac{ \overline{ a_{22}}}{ \overline{ b_{22}} + \exp (\varrho _{2}) } + \frac{\overline{ a_{21}}}{ \overline{ b_{21}} + \exp (\varrho _{1})} \exp (\varrho _{1}-\varrho _{2} ) - \overline{ \mathcal{H}_{2}} \\ \quad{} + \overline{ c_{2}} \exp ( - \overline{ \delta _{2}} \exp (\varrho _{2}) )=0, \end{cases}\displaystyle \end{aligned}$$

satisfies $\|(\varrho _{1}, \varrho _{2})\| = |\varrho _{1}| + |\varrho _{2}|< S$ and $\max | \varrho _{1}(\nu )| + \max |\varrho _{2}(\nu )| < S$. Define a set as follows:

$$\begin{aligned} \mathcal{O} = \bigl\{ \bigl(\varrho _{1}(\nu ), \varrho _{2}(\nu )\bigr)^{T}\in \mathcal{Z}: \bigl\Vert (\varrho _{1}, \varrho _{2})^{T} \bigr\Vert < S \bigr\} . \end{aligned}$$

This satisfies condition (I) in Lemma 1. If

$$\begin{aligned} \varrho \in \partial \mathcal{O} \cap \ker \mathscr{L}= \partial \mathcal{O} \cap \mathbb{R}^{2}, \end{aligned}$$

then *ϱ* is a constant vector in $\mathbb{R}^{2}$ with $\| \varrho \|=S$ satisfying

$$\begin{aligned} \mathscr{Q}\mathscr{N}(\varrho _{1}, \varrho _{2} ) = \begin{pmatrix} - \frac{ \overline{ a_{11}}}{ \overline{ b_{11}} + \exp (\varrho _{1}) } + \frac{ \overline{a_{12}} }{ \overline{ b_{12}} + \exp (\varrho _{2}) } \exp ( \varrho _{2} -\varrho _{1} ) \\ - \overline{ \mathcal{H}_{1} } + \overline{ c_{1}} \exp (- \overline{ \delta _{1}} \exp (\varrho _{1}) ) \\ - \frac{ \overline{a_{22}}}{ \overline{ b_{22}} + \exp (\varrho _{2}) } + \frac{ \overline{ a_{21}}}{ \overline{ b_{21}} + \exp ( \varrho _{1}) } \exp ( \varrho _{1}-\varrho _{2} ) \\ - \overline{ \mathcal{H}_{2} } + \overline{ c_{2}} \exp (- \overline{\delta _{2}}e^{\varrho _{2}} ) \end{pmatrix} \neq \begin{pmatrix} 0 \\ 0 \end{pmatrix}. \end{aligned}$$

Therefore condition (II) is satisfied in Lemma 1. In order to verify condition (III) in Lemma 1, we consider a homotopy

$$\begin{aligned} B_{\mu } \bigl( (\varrho _{1}, \varrho _{2})^{T} \bigr) = \mu J \mathscr{Q}\mathscr{N} \bigl( (\varrho _{1}, \varrho _{2})^{T} \bigr) + (1-\mu ) \rho \bigl( (\varrho _{1}, \varrho _{2} )^{T} \bigr). \end{aligned}$$

By a direct computation and the invariance property of homotopy, one has

$$\begin{aligned} &\deg \bigl( J \mathscr{Q} \mathscr{N} (\varrho _{1}, \varrho _{2})^{T}, \Omega \cap \ker \mathscr{L}, (0,0)^{T} \bigr) \\ &\quad = \deg \bigl( \rho (\varrho _{1}, \varrho _{2})^{T}, \mathcal{O} \cap \ker \mathscr{Q}, (0,0)^{T} \bigr) \neq 0. \end{aligned}$$

Hence Ω verifies all the requirements in Lemma 1. Then we get that equation (4) has at least one periodic solution $(\varrho _{1}, \varrho _{2})^{T}$ with period *p* in $\operatorname{Dom } \mathscr{Q} \cap \mathcal{O}$, which implies that system (1) has at least one positive periodic solution

$$\begin{aligned} \bigl(\exp (\varrho _{1}), \exp (\varrho _{2}) \bigr)^{T}, \end{aligned}$$

with period *p*. The proof is completed. □

## An example for the system

Now, we illustrate the main Theorem 3 with the following model.

### Example 1

**Figure 1:**
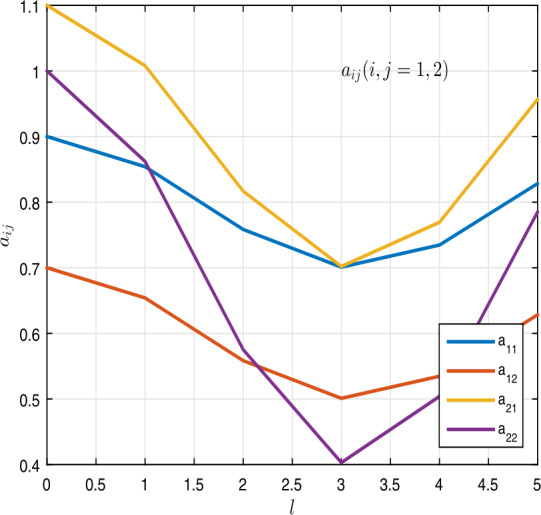
Graphics of $a_{ij}$, $i, j=1,2$, in Example 1

**Figure 2:**
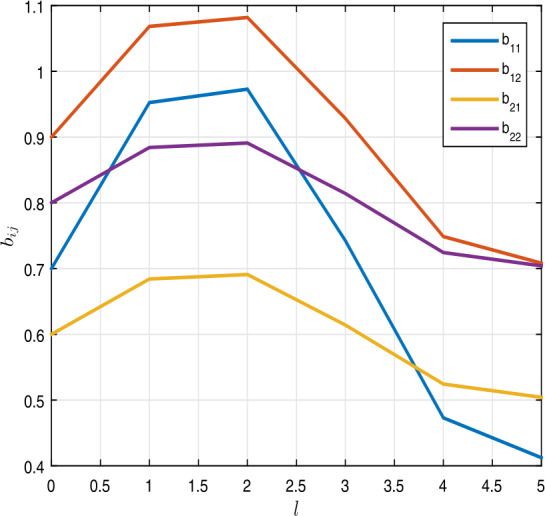
Graphics of $b_{ij}$, where $i, j =1,2$, in Example 1

Consider that a discrete Nicholson’s dual system similar to system (1)

25 $$\begin{aligned} \textstyle\begin{cases} k_{1}(\nu +1) = k_{1}(\nu ) \exp ( - \frac{ 0.8 +0.1 \cos \nu }{ 0.7 + 0.3 \sin \nu + k_{1} ( \nu ) } \\ \phantom{k_{1}(\nu +1) =} {} + \frac{ 0.6 + 0.1 \cos \nu }{ 0.9 + 0.2 \sin \nu + k_{2}(\nu ) } \frac{k_{2}(\nu )}{ k_{1}(\nu ) } \\ \phantom{k_{1}(\nu +1) =}{} -( 2.2 + \cos \nu ) \frac{ k_{1}( \nu - (0.8 + 0.2\cos \nu ))}{ k_{1}(\nu ) } \\ \phantom{k_{1}(\nu +1) =}{} + \frac{( 1.6 + 0.3 \cos \nu ) k_{1}( \nu - (0.8 + 0.2\cos \nu )) }{ k_{1}(\nu ) } \\ \phantom{k_{1}(\nu +1) =}{} \times \exp ( - ( 1 + \frac{ \sin \nu }{ 2} ) k_{1} ( \nu - (0.8 + 0.2\cos \nu ) ) ) ), \\ k_{2}(\nu +1) = k_{2}(\nu ) \exp ( - \frac{ 0.7 + 0.3 \cos \nu }{ 0.8 + 0.1\sin \nu + k_{2}(\nu )} \\ \phantom{k_{2}(\nu +1) = }{} + \frac{0.9 + 0.2\cos \nu }{ 0.6 + 0.1 \sin \nu + k_{1}(\nu )} \frac{k_{1}(\nu )}{ k_{2}(\nu )} \\ \phantom{k_{2}(\nu +1) = }{} -( 3 - 0.1 \sin \nu ) \frac{ k_{2} ( \nu -( 0.6 + 0.1 \sin \nu ) )}{ k_{2}(\nu ) } \\ \phantom{k_{2}(\nu +1) = }{} + \frac{( 1.4 + 0.1 \cos \nu ) k_{2} ( \nu - ( 0.6 + 0.1 \sin \nu ) ) }{ k_{2}(\nu )} \\ \phantom{k_{2}(\nu +1) = }{} \times \exp (- ( 1 + \frac{\cos \nu }{2} ) k_{2} (\nu -(0.6+0.1\sin \nu )) ) ), \end{cases}\displaystyle \end{aligned}$$

has at least one 2*π*-periodic solutions. Clearly, $I_{p}=\{ 0, 1, 2, \ldots,19, 20 \}$ and

$$\begin{aligned} &a_{ij}(l) = \begin{bmatrix} 0.8 +0.1 \cos l & 0.6 + 0.1 \cos l \\ 0.9 + 0.2\cos l & 0.7 + 0.3 \cos l \end{bmatrix}, \\ &b_{ij}(l) = \begin{bmatrix} 0.7 + 0.3 \sin l & 0.9 + 0.2 \sin l \\ 0.6 + 0.1 \sin l & 0.8 + 0.1 \sin l \end{bmatrix}, \\ &c_{i}(\nu ) = \begin{bmatrix} 1.6 + 0.3\cos \nu \\ 1.4+ 0.1 \cos \nu \end{bmatrix},\qquad \tau _{i}(\nu ) = \begin{bmatrix} 0.8 + 0.2\cos \nu \\ 0.6 + 0.1 \sin \nu \end{bmatrix}, \\ &\delta _{i}(\nu ) = \begin{bmatrix} 1 + \frac{ \sin \nu }{ 2} \\ 1+\frac{\cos \nu }{2} \end{bmatrix},\qquad \mathcal{H}_{i}( \nu ) = \begin{bmatrix} 2.2 + \cos \nu \\ 3.0 - 0.1 \sin \nu \end{bmatrix} \end{aligned}$$

for $i, j=1, 2$. Table 1 shows the results of $a_{ij}$ and $b_{ij}$, where $i=1,2$ and $j=1,2$. Also, one can see the graphs of $a_{ij}$ and $b_{ij}$ in Figs. 1 and 2. In addition, for $\nu \in I_{p}$, we get

$$\begin{aligned} &a_{11}^{\ast } = 0.9000,\qquad a_{12}^{\ast } = 0.7000,\qquad a_{21}^{ \ast } = 1.1000,\qquad a_{22}^{\ast } = 1.0000, \\ &{b_{11}}_{\ast } = 0.4123,\qquad {b_{12}}_{\ast } = 0.7082, \qquad{b_{21}}_{ \ast } = 0.5041,\qquad {b_{22}}_{\ast } = 0.7041. \end{aligned}$$

**Table 1:** Numerical results of $a_{ij}$ and $b_{ij}$, where $i=1,2$ and $j=1,2$, in Example 1

*ν*	$a_{ij}$				$b_{ij}$			
	$a_{11}$	$a_{12}$	$a_{21}$	$a_{22}$	$b_{11}$	$b_{12}$	$b_{21}$	$b_{22}$
0	0.9000	0.7000	1.1000	1.0000	0.7000	0.9000	0.6000	0.8000
1	0.9000	0.7000	1.1000	1.0000	0.7005	0.9003	0.6002	0.8002
2	0.9000	0.7000	1.1000	1.0000	0.7010	0.9007	0.6003	0.8003
3	0.9000	0.7000	1.1000	1.0000	0.7016	0.9010	0.6005	0.8005
4	0.9000	0.7000	1.1000	1.0000	0.7021	0.9014	0.6007	0.8007
5	0.9000	0.7000	1.1000	1.0000	0.7026	0.9017	0.6009	0.8009
6	0.9000	0.7000	1.1000	1.0000	0.7031	0.9021	0.6010	0.8010
7	0.9000	0.7000	1.1000	1.0000	0.7037	0.9024	0.6012	0.8012
8	0.9000	0.7000	1.1000	1.0000	0.7042	0.9028	0.6014	0.8014
9	0.9000	0.7000	1.1000	1.0000	0.7047	0.9031	0.6016	0.8016
10	0.9000	0.7000	1.1000	1.0000	0.7052	0.9035	0.6017	0.8017
11	0.9000	0.7000	1.1000	0.9999	0.7058	0.9038	0.6019	0.8019
12	0.9000	0.7000	1.1000	0.9999	0.7063	0.9042	0.6021	0.8021
13	0.9000	0.7000	1.0999	0.9999	0.7068	0.9045	0.6023	0.8023
14	0.9000	0.7000	1.0999	0.9999	0.7073	0.9049	0.6024	0.8024
15	0.9000	0.7000	1.0999	0.9999	0.7079	0.9052	0.6026	0.8026
16	0.9000	0.7000	1.0999	0.9999	0.7084	0.9056	0.6028	0.8028
17	0.9000	0.7000	1.0999	0.9999	0.7089	0.9059	0.6030	0.8030
18	0.9000	0.7000	1.0999	0.9999	0.7094	0.9063	0.6031	0.8031
19	0.8999	0.6999	1.0999	0.9998	0.7099	0.9066	0.6033	0.8033

Now, by using (3), we obtain

$$\begin{aligned} \mathcal{H}_{1}^{\ast } = 3.5000, \qquad\mathcal{H}_{2}^{\ast } = 3.0958,\qquad {\mathcal{H}_{1}}_{\ast } = 1.2100,\qquad { \mathcal{H}_{2}}_{ \ast } = 2.9090. \end{aligned}$$

In this level, the numerical results in Table 1 imply that

$$\begin{aligned} \frac{a_{11}^{\ast }}{ b_{11}}_{ \ast } + \mathcal{H}_{1}^{\ast } = 5.3827>0, \qquad\frac{a_{22}^{ \ast }}{b_{22}}_{\ast } + \mathcal{H}_{2}^{\ast } = 4.5161>0, \end{aligned}$$

and

$$\begin{aligned} {\mathcal{H}_{1}}_{\ast } = 1.2100 > 0.9884= \frac{a_{12}^{ \ast }}{{ b_{12}}_{\ast }}, \qquad {\mathcal{H}_{2}}_{ \ast } = 2.9090> 2.1820 = \frac{a_{21}^{ \ast }}{{b_{21}}_{\ast }}. \end{aligned}$$

By employing Algorithms 1 and 2, we can compute the above results.

Thus assumptions (1), (2), and (3) all hold for system (25) from the main Theorem 3. Thus, Theorem 3 yields that system (25) has at least one 2*π*-periodic solution.

## Conclusions and discussion

In the last decades, Nicholson’s blowflies model has found successful applications in many areas such as population dynamics, system control theory, biomathematics, and optimization problems. In this paper, we study a discrete Nicholson’s dual system with density-dependent morality harvesting terms. Some sufficient conditions for the existence of positive periodic solutions have been established. Moreover, a numerical example is given to show the feasibility of our results. Also, this result relates to biological modeling [5, 16, 59].

## Figures and Tables

**Algorithm 1:**
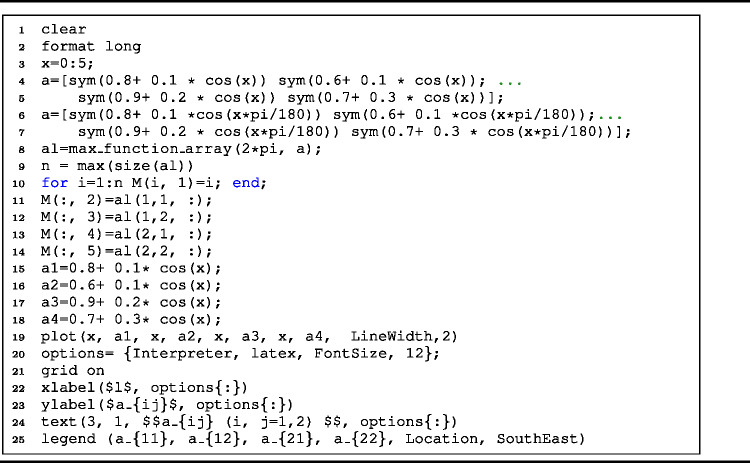
The MATLAB lines for computing $a_{ij}(l)$

**Algorithm 2:**
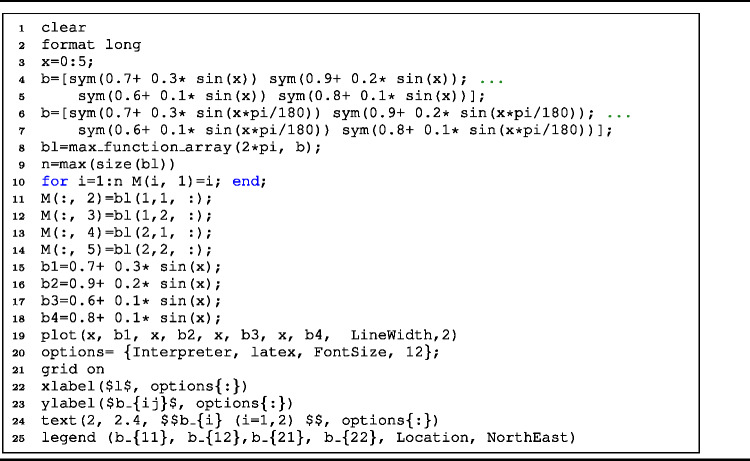
The MATLAB lines for computing $b_{ij}(l)$

## Data Availability

Data sharing not applicable to this article as no datasets were generated or analyzed during the current study.
